# How to use three-dimensional optical coherence tomography effectively in coronary bifurcation stenting

**DOI:** 10.3389/fcvm.2022.1023834

**Published:** 2022-11-11

**Authors:** Yoshinobu Murasato

**Affiliations:** Department of Cardiology and Clinical Research Institute, National Hospital Organization Kyushu Medical Center, Fukuoka, Japan

**Keywords:** optical coherence tomography, three-dimensional image, coronary bifurcation, drug-eluting stent, guidewire

## Abstract

Imaging-guided coronary bifurcation intervention has improved clinical outcomes due to the appropriate size selection of the devices and optimization of the procedure (sufficient stent expansion, reduction of stent malapposition, appropriate stent landing zone, and detection of vessel dissection). In particular, three-dimensional optical coherence tomography (3D OCT) facilitates clear visualization of stent configuration and guidewire position, which promotes optimal guidewire crossing to the side branch. Successive side branch dilation leads to wide ostial dilation with less strut malapposition. However, the link connection of the stent located on the bifurcated carina has been found to be an impediment to sufficient opening of the side branch, resulting in incomplete strut apposition. In such cases, the aggressive proximal optimization technique improves the jailing strut pattern, and 3D OCT navigates the guidewire crossing to the optimal cell that is most likely to be expanded sufficiently, which is not always a distal cell. In two-stent deployment, 3D OCT facilitates optimal guidewire crossing, which leads to less metallic carina, clustering, and overlapping. The present review describes a method of clear visualization and assessment with 3D OCT and discusses the efficacy of 3D OCT in coronary bifurcation stenting in clinical practice.

## Introduction

Intracoronary imaging guidance provides an optimal procedure for device selection, stent landing zone, expansion, and apposition in coronary bifurcation interventions, which can improve clinical outcomes (1). Optical coherence tomography (OCT) can provide higher-resolution analysis compared to intravascular ultrasound, which facilitates a more accurate assessment of plaque characteristics, vessel lumen, stent expansion, and apposition (1–3). Particularly in coronary bifurcation intervention, a realistic three-dimensional (3D) reconstruction of stent configuration during or after complex procedures and guide-wire recrossing to the distally located cell in the side branch (SB) are effective in promoting optimal SB dilation with less stent malapposition or deformation (1, 3–8). In this article, studies on the efficacy of 3D OCT in coronary bifurcation are reviewed, and its practical use is clarified, indicating several useful cases.

## Assessment of policy and implications

### Method of 3D OCT imaging

For clear visualization in OCT imaging, complete removal of red blood cells from the vessel by flushing is required. In the OCT-guided coronary bifurcation intervention, five or more observations are recommended, which include pullbacks from both the main vessel (MV) and SB in pre-intervention and at the final procedure, assessment of guidewire recrossing to the SB, and correction of stent failure or wiring failure (3, 7, 9–12). To reduce the consumption of contrast medium, manual flushing with 7–15 mL of low-molecular-weight dextran (LMWD) can be used for repeated observations. Although renal toxicity of LMWD has been reported in systematic administration to stabilize blood pressure (13), the small amount used for flushing in the OCT observation does not have a significant effect on renal function due to total consumption of <100 mL (9, 14). The image quality obtained after LMWD flushing is similar to that obtained after flushing with a contrast medium and is sufficient to reconstruct a 3D image (15) (Supplementary Movie 1). Online 3D imaging reconstruction is available in two types of frequency-domain OCT machines (ILUMIEN Optis; Abbott Vascular, Westford, MA, USA) and optical coherence frequency-domain imaging (OFDI; Lunawave; Terumo Corp., Tokyo, Japan). After autodetection of the stent and vessel surface, a stent-enhanced view of the 3D vessel and its fly-through view are available. The carpet view is optionally available for OFDI.

### Advantage of 3D OCT imaging in coronary bifurcation intervention

**Figure 1:**
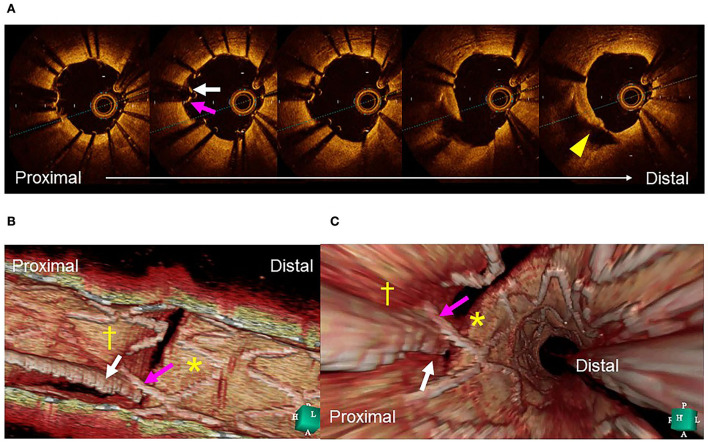
Assessment of guidewire recrossing point before side branch (SB) dilation. **(A)** Two-dimensional optical coherence tomography image. The guide wire (arrow) recrossed at the edge of the SB away from its center (triangle). **(B)** Three-dimensional image clearly demonstrates the guidewire (arrow) recrossing in the distal part of the proximal cell indicated by cross (†) and not in the optimal distal cell indicated by asterisk (*). **(C)** Fly-through image also shows suboptimal wiring (arrow) into the proximal cell (†) and not in the distal cell (*) (Supplementary Movie 2).

**Figure 2:**
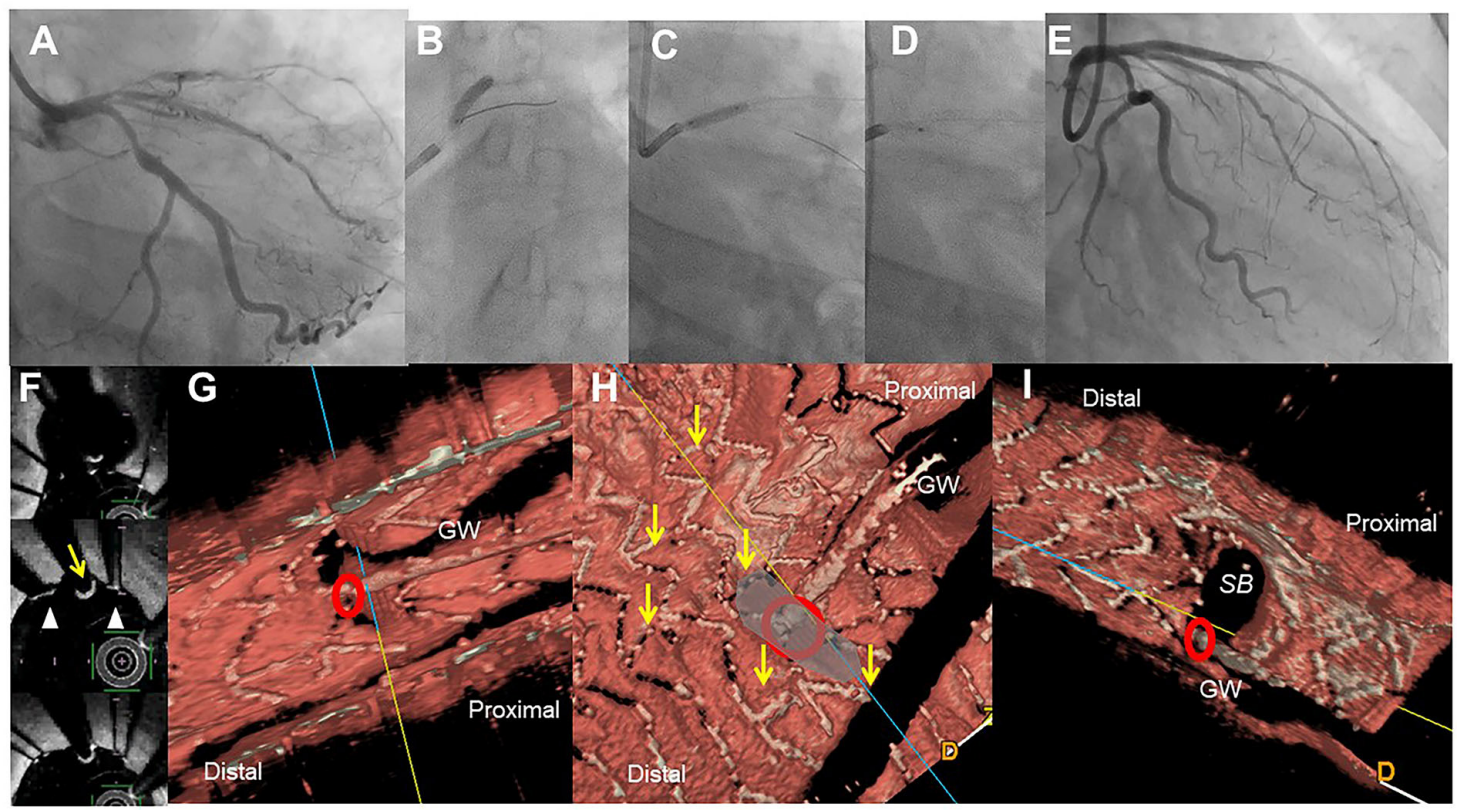
Assessment of guide wire recrossing point in the ostium before side branch (SB) dilation in the provisional stenting. A 51-year-old woman with Medina 1-1-1 lesion in left anterior descending artery (LAD)-diagonal bifurcation **(A)** was treated with crossover stenting, 3.0/28 mm Xience Alpine, **(B)** followed by proximal optimization technique **(C)** and SB dilation **(D)**, which resulted in acceptable results **(E)** (Supplementary Movie 3). Two-dimensional optical frequency domain imaging in the bifurcation (**F**, top to bottom; pull back images from the distal) shows that the guidewire recrossed distally (yellow arrow) between the struts (triangles) at the carina. Three-dimensional vessel view **(G)** and carpet view **(H)** clearly demonstrates the guidewire recrossing point (red circle) with no link connection (yellow arrows) in the SB ostium. Final three-dimensional image shows wide opening of the SB without any jailing struts **(I)**.

Clear visualization of the jailing strut configuration and guidewire recrossing in the SB in the 3D OCT image facilitates optimal and more accurate guidewire recrossing (4–6, 8). In the first guidewire recrossing to the SB after MV stenting under angio-guidance, the success rate of optimal distal cell wiring is 55–66% (4–6, 8), and can be elevated to 87–100% after correction of the guidewire recrossing under 3D OCT guidance (4–6, 8). Since the first success rate in the left main (LM) bifurcation is particularly low at 55%, 3D OCT-guided guidewire recrossing results in less incomplete strut apposition (ISA) in the LM bifurcation compared to 2D OCT guidance, in which some wiring failures are ignored (10.3 ± 8.9% vs. 18.7 ± 12.8%, *P* = 0.014) (5). Although an assessment of the guidewire recrossing point is feasible in 2D imaging, it requires meticulous frame-by-frame confirmation of the absence of metal struts overlapping the guidewire at the carina. However, accurate assessment remains difficult owing to the complex shape of the stent cells. As shown in Figure 1, there is a strut overlapping the guidewire at the carina (pink arrow), but its location is in the rim of the SB in the 2D OCT image (A). 3D OCT imaging clearly demonstrates that the guidewire recrossing point is located in the distal site of the cell located proximally in the SB ostium (white arrow in the cell indicated by cross [†]) and suggests that the neighboring distal cell (indicated by asterisk [^*^]) is optimal for guidewire recrossing (B and C) (Supplementary Movie 2). In the case of a 1-1-1 bifurcation lesion shown in Figure 2 (Supplementary Movie 3), the 2D OFDI image indicates no strut overlapping the guidewire at the carina, suggesting optimal wiring (F), and the 3D OFDI vessel view (G) and carpet view (H) more clearly demonstrate the jailing strut configuration and location of the guidewire recrossing point and link connection, which facilitates the confirmation of the optimal wiring and subsequent optimal SB dilation (I).

### Guidewire recrossing cell and jailing strut configuration on the SB ostium

**Figure 3:**
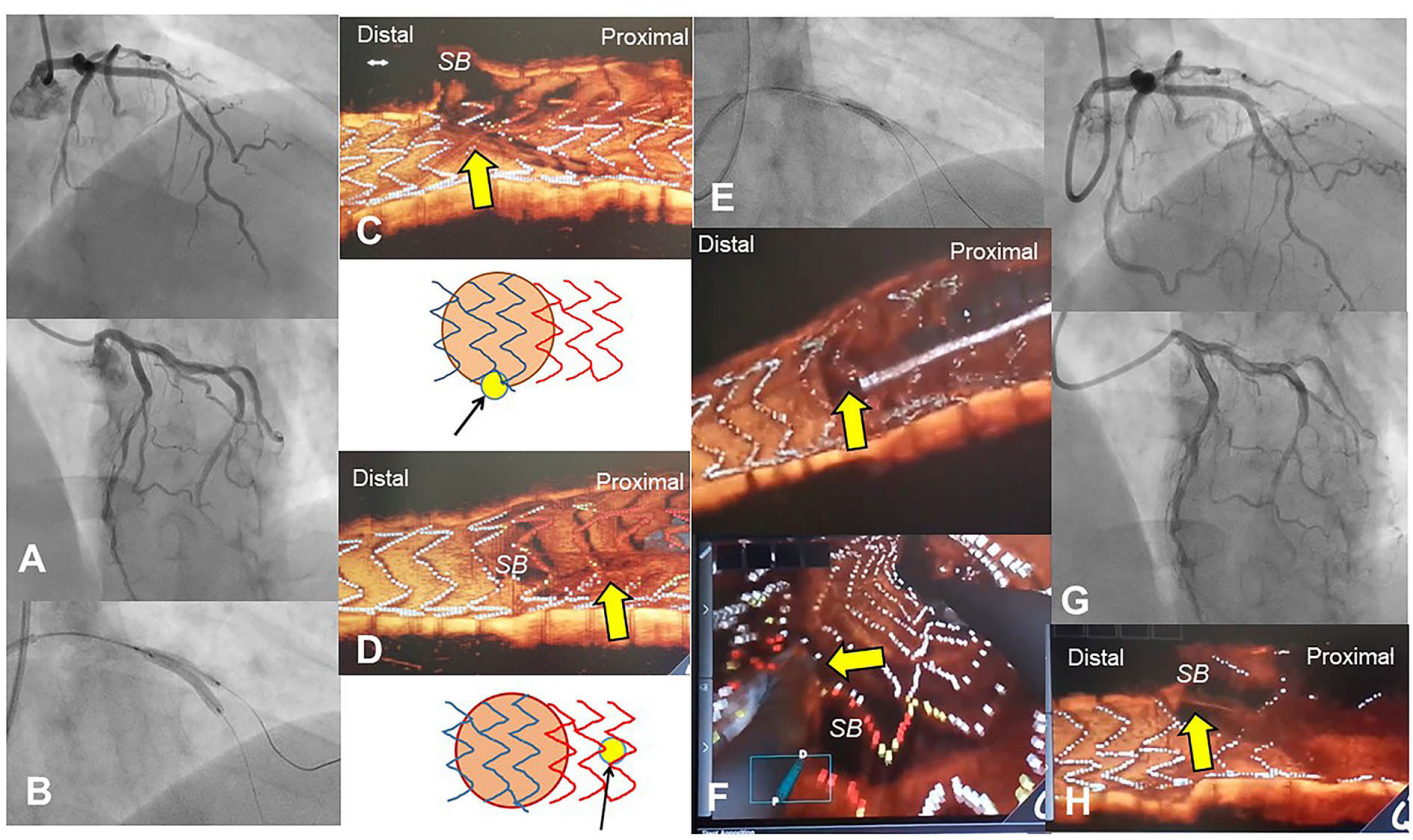
Optimal guidewire recrossing using 3D optical coherence tomography (OCT) imaging after jailed balloon technique. A 55-year-old man with Medina 1-1-1 lesion in left anterior descending artery (LAD)-diagonal bifurcation **(A)** was treated with crossover stenting with jailed balloon technique that accompanied simultaneous dilation of a 2.75/18 mm Ultimaster stent and a jailed 2.0 mm balloon **(B)** (Supplementary Movie 4). Since the proximal part of the jailed balloon protruded in the LAD, significant stent malapposition around the ostium of the diagonal branch inhibits optimal guidewire recrossing. First guidewire recrossing is in the rim of the ostium and outside of the link connection, which was suboptimal (**C**, arrow) (Supplementary Movie 5). Second guidewire recrossing is in a more proximal cell located outside of the ostium, which is also suboptimal (**D**, arrow) (Supplementary Movie 5). After proximal optimization with 2.75 mm balloon **(E)**, guidewire recrossing in the optimal distal cell is confirmed in the 3D OCT imaging (**F** top: 3D vessel view, bottom: 3D fly-through view). After final kissing balloon inflation (KBI) with 2.75 and 2.0 mm balloons, final coronary angiography shows acceptable results **(G)** (Supplementary Movie 4). The 3D OCT shows a wide opening of the diagonal branch ostium without any stent deformation or malapposition **(H)** (Supplementary Movie 5).

Previous bench testing has revealed that guidewire recrossing in the most distal cell among the jailing cells on the SB ostium provides the wide opening of the SB with less ISA in the SB dilation after MV stenting, while guidewire recrossing in the proximal cell creates an inverse protrusion of the jailing cells into the MV lumen, which remains as a metallic carina (16, 17). Recent 3D OCT studies revealed the significant impact of the presence of a link connection of the stent located at the carina on ISA and optimal distal wiring (4, 5, 18). The jailing strut configuration pattern is divided into two types according to the presence of link connection at the carina: link-free (LF) type and link-connecting (LC) type (4–6, 11, 18) (Figures 3A,B). Cases of the LF type that achieve optimal distal wiring have less ISA compared to suboptimal wiring in the LF type (4, 11). In contrast, even though optimal distal wiring can be achieved in cases of the LC type, a significant reduction in ISA may not be obtained due to the limitation of cell expansion or incomplete removal of the link connection from the SB ostium (4, 11). As the routine SB dilation, including final kissing balloon inflation (KBI), has not been recommended (19, 20), SB dilation for removing the jailing struts is not necessary when the SB jailing is not significant (no or less jailing [NLJ] type) (Figure 3), such as in the cases that the number of jailing struts is less than three or the area surrounded by the jailing struts is <25% of the SB ostium area. Abluminal wiring, in which the guidewire partially or entirely recrosses outside the stent through the SB, leads to serious stent deformation (21) and 3D OCT imaging contributes to its detection. Figure 3 shows a case of a 1-1-1 bifurcation lesion treated with crossover stenting and the jailed-balloon technique (22) for protection from SB occlusion (Supplementary Movie 4). The first and second guidewires recrossed laterally and proximally to the SB ostium, resulting in abluminal wiring due to significant stent malapposition caused by the inflation of the jailed balloon. After a proximal optimization technique (POT) with an optimal-sized balloon (21), the optimal guide wire recrossing to the distal cell was promoted and confirmed in the 3D OCT images (Supplementary Movie 5). Adequate SB dilation, without significant stent deformation, was achieved.

### Efficacy of two-stent deployment

Provisional 2-stentings after insufficient SB treatment following MV stenting are culotte, T-stenting and minimal protrusion (TAP), and T-stenting. The proximal ends of the SB stents are located proximally across the main branch, minimally protruded in the carina, and on the SB ostium, respectively. The guidewire recrossing to both MV and SB is required in culotte stenting. Although the guidewire recrossing is only needed in the SB, TAP, and T-stenting result in some metallic carina formation or have a risk of gap formation between MV and SB stents. Elective 2-stenting which SB stenting is prior to MV stenting are crush, DK-crush, culotte, and T-stenting. In crush and DK-crush stentings, the proximal part of the SB stent is crushed by a balloon simultaneously located in the MV, and it is followed by final KBI alone after MV stenting in crush stenting and two-time KBI after crushing SB stent and at final in DK-crush stenting. The guidewire recrossing is required before KBI in both crush stentings, which are one and two times, respectively. In culotte stenting, the SB stent is deployed proximally to the main branch and guidewire recrossing is required two times, similarly to the provisional approach. In T-stenting, guide wire recrossing is not necessary; however, in case of more protrusion of the SB stent into the MV, a similar procedure as in crush stenting is necessary for the complete removal of the jailing struts.

**Figure 4:**
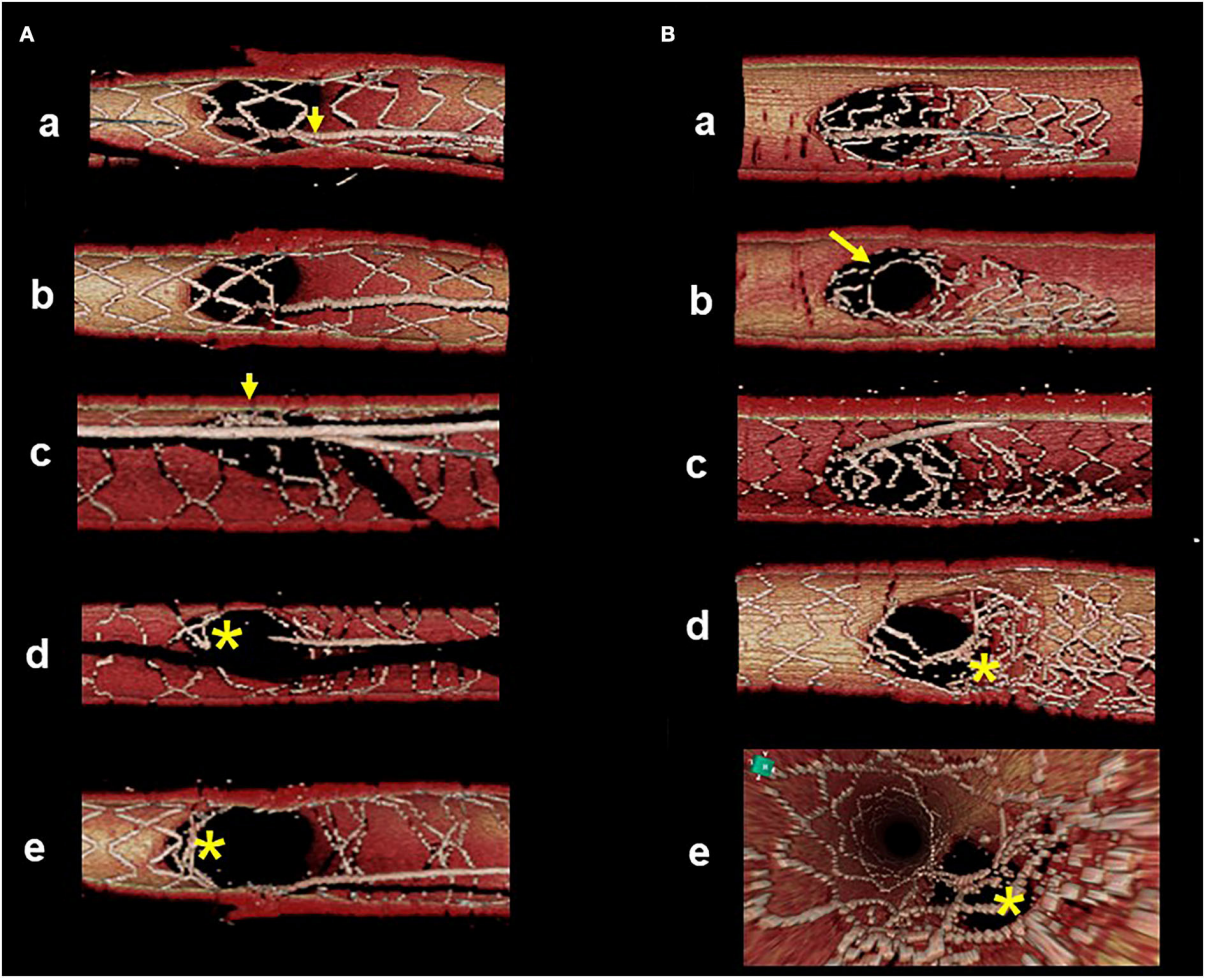
Suboptimal guidewire recrossing in 2-stenting under angio-guidance. 3D optical coherence tomography (OCT) imaging was *post-hoc* analysis. **(A)** Culotte stenting. First guidewire recrossing to the main vessel (MV) is in the proximal cell (a, arrow) and subsequent MV dilation left jailing struts at the carinal site (b). After MV stenting, second guidewire recrossing to the side branch (SB) is optimal in the distal cell (c, arrow). Even after final kissing balloon inflation (KBI), metallic carina remains as the asterisk indicates (d: view from MV, e: view from SB). **(B)** Double kissing (DK)-crush stenting. After SB stent crush, first guidewire recrossing to the middle part of SB (a). First KBI made the hole in the proximal site (b). After MV stenting, second guidewire recrossing outside of the hole made by first KBI (c, recrossing point is indicated by the arrow in **b**). Even after second KBI, distorted struts are clustered in the proximal part of the SB ostium, indicated by the asterisk (d: view from MV, e: view from proximal to the bifurcation).

**Figure 5:**
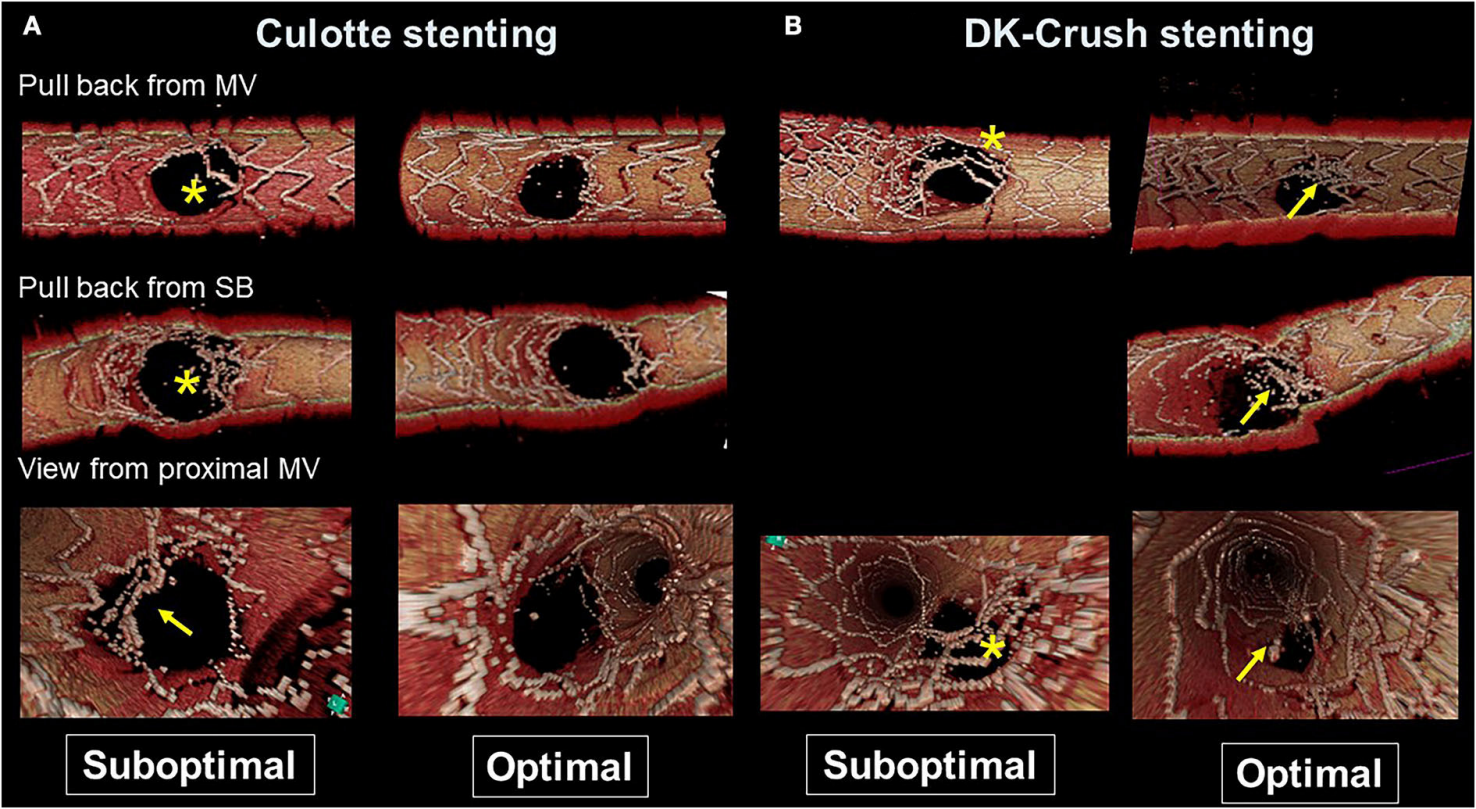
Representative optimal and suboptimal cases of 2-stent on 3D optical coherence tomography (OCT) assessment. **(A)** Culotte stenting. In a suboptimal case (left column), metallic carina remains (asterisks in pullback views from the main vessel [MV] and side branch [SB]) and there is insufficient stent expansion in the main branch ostium with malapposed strut at the lateral site (arrow in the view from proximal MV). In an optimal case (right column), both branch ostia are well-expanded with good apposition. **(B)** Double kissing (DK)-crush stenting. In a suboptimal case (left column) due to suboptimal wiring outside of the hole made by first kissing balloon inflation (KBI), jailing struts remain at the SB ostium (asterisks). In an optimal case (right column), metallic carina remains (arrows), but it is located in a neutral position between both branches and creates no limitation for MV stent expansion or incomplete stent crush.

**Figure 6:**
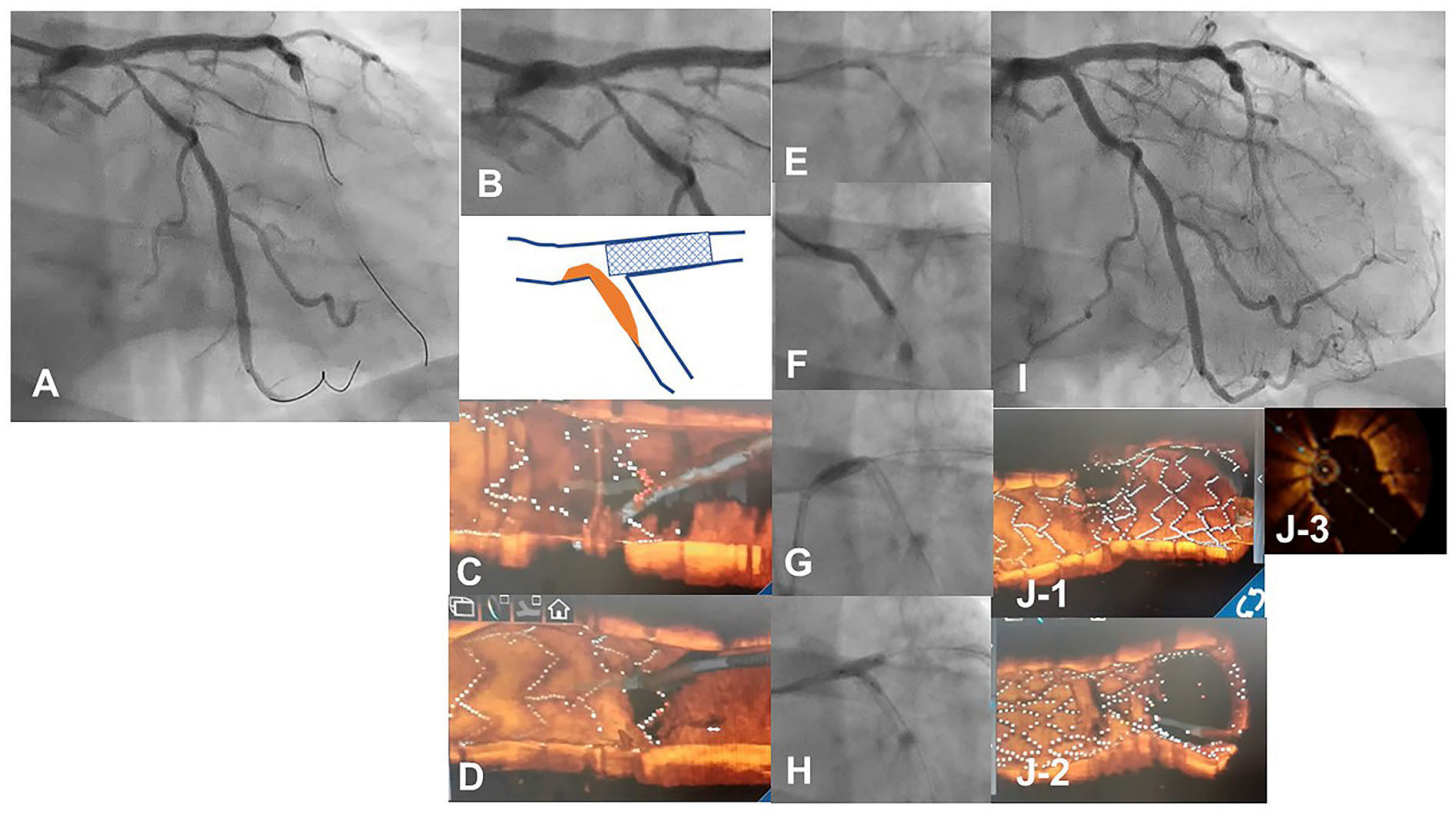
3D optical coherence tomography (OCT) guided guidewire crossing in a one-string culotte stenting. A 63-year-old man who underwent a 3.5/28 mm Xience Alpine stent implantation for chronic total occlusion of the proximal left anterior descending artery (LAD) previously was treated for the progression of stenosis in left main (LM) to the left circumflex artery (LCX) **(A)**. Medina 1-0-1 lesion in the LM bifurcation **(B)** was identified (Supplementary Movie 6) and LAD stent was minimally protruded in the LCX ostium (**B** bottom scheme). First guidewire crossing to the most proximal cell of the LAD stent failed **(C)** and second crossing using a double-lumen catheter succeeded **(D)** (Supplementary Movie 7). After the dilation of the cell to one string using a 2.5 mm balloon **(E)**, a 3.0/26 mm resolute integrity stent was deployed from LM to proximal LCX **(F)** to perform one-string culotte stenting. After proximal optimization using a 4.0 mm balloon **(G)**, guidewire recrossing to the LAD in the optimal cell is confirmed in the 3D OCT imaging and final KBI using 4.0 and 2.5 mm balloons is completed **(H)**. Final coronary angiography shows acceptable results **(I)** (Supplementary Movie 6), and OCT images show adequate dilation without significant incomplete strut apposition in both branch ostia and one-string metal overlapping in the LM (**J−1**: pullback from LCX, **J-2**: pullback from LAD, **J-3**: 2D image of the bifurcation in the pullback from LCX) (Supplementary Movie 8).

**Figure 7:**
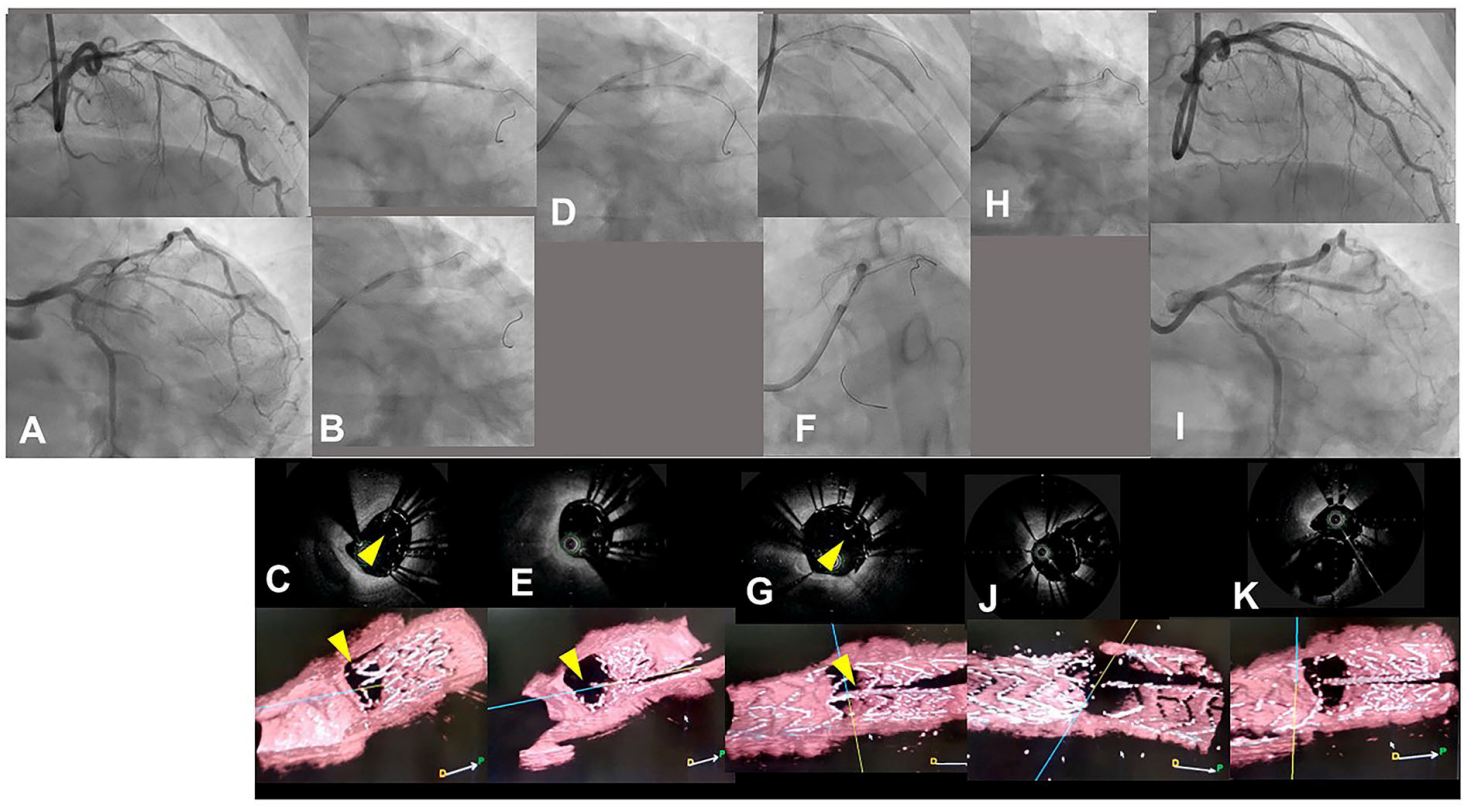
Assessment of guidewire recrossing point in double kissing (DK)-crush stenting with three-dimensional optical coherence frequency-domain imaging (OFDI). A 68-year-old man with Medina 0-1-1 lesion in left anterior descending artery (LAD)-diagonal bifurcation **(A)** (Supplementary Movie 9) was treated with DK-crush stenting. After a Ultimaster 2.25/38 mm stent was implanted in diffusely diseased diagonal branch with 3 mm protruded into the LAD (**B** top panel), a 3.0 mm balloon placed in the LAD crushed the proximal site of the side branch stent (**B** bottom panel). The guidewire recrossed inside the crushed stent, which is confirmed with two-dimensional and three-dimensional OFDI images (**C** top and bottom panels, yellow arrowheads: guidewires) (Supplementary Movie 10). First kissing balloon inflation (KBI) using 3.0 mm and 2.25 mm balloons was performed **(D)**. Side branch stent is crushed more in the two-dimensional OFDI image (**E** top panel) and side branch ostium is open wide in the three-dimensional image (**E** bottom panel) (Supplementary Movie 10). Xience Alpine 2.5/28 and 3.0/23 mm stents were implanted in the middle LAD and left main and proximal LAD, respectively (**F** top and bottom panels). The OFDI image shows that the guidewire recrossed in the cell on the hole made by the first KBI (**G**, yellow arrow) (Supplementary Movie 10). Second KBI was performed using 3.0 and 2.25 mm balloons **(H)**. Final coronary angiography shows acceptable results **(I)** (Supplementary Movie 9) and OFDI images show adequate dilation in both branch ostia with minimal metallic carina (**J**: pullback from LAD, **K**: pullback from diagonal branch) (Supplementary Movie 10).

**Figure 8:**
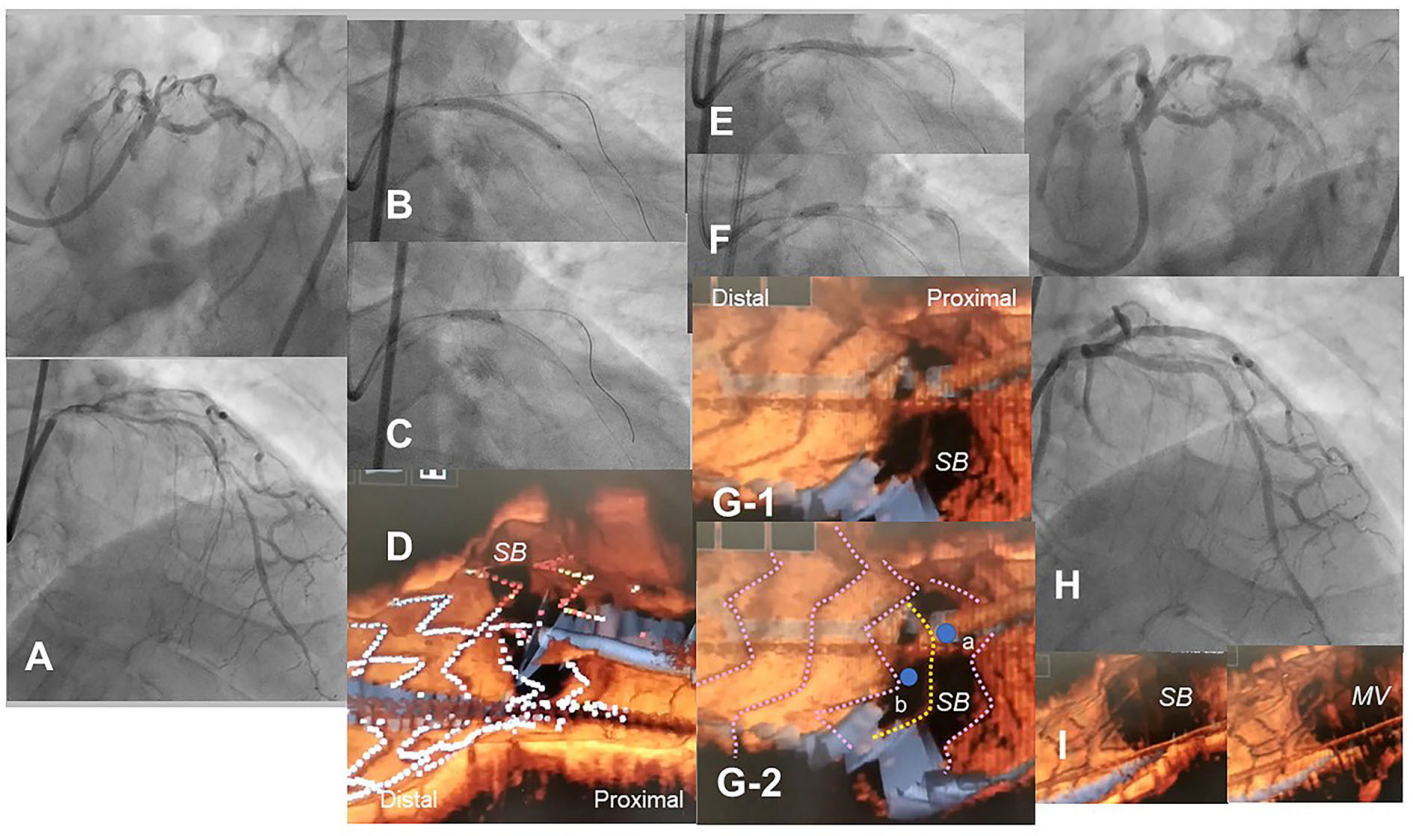
Optical coherence tomography (OCT)-guided culotte stenting after rotational atherectomy in the calcified bifurcation lesion. A 72-year-old man with Medina 0-1-1 calcified lesion in left anterior descending artery (LAD)-diagonal bifurcation **(A)** was treated with culotte stenting after rotational atherectomy in both branches (Supplementary Movie 11). Rotational atherectomy using 1.75 mm and 2.15 mm burrs was performed in both LAD and diagonal branch, and OCT shows adequate ablation. A Ultimaster 3.0/33 mm stent was implanted from proximal LAD to diagonal branch **(B)** followed by proximal optimization with a 3.75 mm balloon **(C)**. 3D OCT showed the guidewire recrossed in the distal cell which occupied most largely in the diagonal branch ostium in the Link-free type that some proximal hoops of the far-distal cells with link-connection remained at the carina **(D)**. After the dilation of diagonal branch ostium, an Ultimaster 3.0/33 mm stent was deployed in the LAD **(E)** followed by proximal optimization with a 3.75 mm balloon **(F)**. The guidewire seemed to recross in the middle part of the jailing cells (**G-2**, point a), and more distal part (**G-1**, point b) seemed to be optimal. However, the strut alignment of the MV stent was indicated as pink lines and the most distal strut indicated by yellow lines was turned out to be the remained hoop of the SB stent at the carina **(G-2)** (Supplementary Movie 12). After confirmation of optimal distal wiring, kissing balloon inflation with two 3.0 mm balloons. Final angiography was acceptable **(H)** and 3D OCT showed no significant stent deformation or malapposition except for minimal remaining the meal hoop **(I)**.

Accurate assessment of guidewire recrossing points and monitoring of actual stent configuration during and after complex procedures are also crucial in two-stent deployment as well as in provisional stenting (16, 17, 23–28). Since it has been shown that angio-guided guidewire recrossing in the optimal cell is only 50–67% in past 3D OCT studies (4–6, 8), 2-stenting requiring multiple guidewires recrossing might be <50% of the success rate of optimal distal cell wiring in all procedures (Table 1). Representative cases treated under angio-guidance are shown in Figure 4, where suboptimal guidewire recrossing in the proximal cell in the main branch ostium resulted in metallic carina formation after culotte stenting (A), and suboptimal guidewire recrossing outside of the hole made by the first KBI led to significant jailing struts remaining after double kissing (DK)-crush stenting (B). In classical and DK-crush stenting, guidewire recrossing to the proximal site of the SB ostium is generally recommended to avoid wiring outside the SB stent, which may cause more crushing of the SB stent (29). The 3D OCT imaging sometimes fails to visualize 2-or 3-layered metal overlapping sites clearly without erroneous recognition of each metal layer and accurate guidewire recrossing point in classical crush stenting; however, clear visualization is obtained after the first KBI in DK-crush stenting due to the reduction of the overlapping layer from the SB ostium. The guidewire is crossed inside the hole made by the first KBI, and one layer of the MV stent on the hole is convenient for observation with 3D OCT imaging. When the guidewire is recrossed outside the hole, whether in proximal or distal sites, the advantage of DK-crush stenting is nullified, and distorted metal clustering occurs in the SB ostium (B). An unfavorable stent configuration was detected in the *post-hoc* analysis of 3D OCT imaging, as shown in Figure 5: ISA in the MV on the side contralateral to the SB and metallic carina formation after culotte stenting (A), and jailing struts remain after the second KBI and metallic carina formation even after guidewire recrossing in the non-distal cell after DK-Crush stenting (B). 3D OCT guidance minimizes metal overlapping, metallic carina formation, and generation of a gap between the stents, and a higher success rate for optimal guidewire crossing leading to a wide opening of both daughter branch ostia (10, 30) (Table 1). These optimal treatments lead to the restoration of normal coronary flow circumstances without the generation of a wide low-shear stress area in the bifurcation (17, 26). One-string culotte stenting (31, 32) is 2-stenting, which ultimately reduces the metal overlap. In the case of failure of stenting in the left anterior descending artery (LAD) alone with strut protrusion in the ostium of the left circumflex artery (LCX), 3D OCT imaging was effective in completing one-string culotte stenting (Figure 6, Supplementary Movie 6). On fluoroscopy, it was difficult to confirm guidewire recrossing in the optimal cell, which was the most proximal cell of the stent previously implanted in the LAD. The first attempt failed, and the guidewire crossed outside the LAD stent. Successful second wire crossing more distal to the first wire was confirmed by stacking the catheter at the jailing struts (33) and 3D OCT imaging (Supplementary Movie 7). The most proximal cell was stretched to a single string and LM-LCX stenting was performed. After confirmation of the optimal guidewire recrossing to the LAD, the final KBI was completed (Supplementary Movie 6). Final OCT images show adequate expansion of the LAD and LCX ostium without significant strut malapposition or any jailing strut on the branch ostium (Supplementary Movie 8). As shown in Figure 7, complex true bifurcation lesions with diffuse stenosis in both the LAD and diagonal branches were treated with DK-crush stenting (Supplementary Movie 9). A long stent was deployed from the proximal LAD to the diagonal branch, and its proximal site was crushed with a balloon placed in the LAD. The guidewire recrossing through the inside of the distal cell of the SB stent was confirmed on 3D OCT imaging. First, KBI was performed and an MV stent was implanted. The guidewire recrossing in the distal cell inside the hole made by the first KBI was confirmed using 3D OCT. The second KBI and final POT were performed, and the final OCT showed adequate expansion of the SB ostium, with minimal metallic carina formation (Supplementary Movie 10) with acceptable angiographical results (Supplementary Movie 9). In 2-stenting, monitoring stent configuration during the procedure with the 3D OCT system is helpful to minimize ISA and stent deformation. As shown in Figure 8, a severely calcified true bifurcation lesion between the LAD and the diagonal branch (Supplementary Movie 11) was treated with culotte stenting after rotational atherectomy. After SB stenting, the guidewire recrossed in the distal cell, which occupied most of the SB ostium in the LF type, and some proximal hoops of the far-distal cells with link connection remained at the carina (D) (Supplementary Movie 12). After the dilation of the cells, the MV stent was deployed. The guidewire seemed to recross in the middle section of the jailing cells (G-2, point a), and the more distal part (G-2, point b) seemed to be optimal (Supplementary Movie 12). However, the strut alignment of the MV stent, indicated by pink lines, and the most distal strut, indicated by a yellow line, was a hoop of the SB stent at the carina. Therefore, we confirmed that the guidewire recrossed in the optimal distal cell, and remained inside the hoop of the SB stent at the carina (Supplementary Movie 12). Subsequent KBI was performed (Supplementary Movie 11), and the final OCT image shows no significant stent deformation or malapposition, except for a minimal remaining metal hoop (I). If the guidewire recrossed in the distal cell-like site (G-2, point b), the abluminal position of the guidewire would lead to malapposition or serious deformation of the SB stent at the carinal site.

**Table 1:** Impact of angio-guidance and three-dimensional optical coherence tomography (3D OCT) guidance on optimal guidewire recrossing and other stent failure in coronary bifurcation stenting.

	**Guidewire recrossing site**	**Angio-guidance**			**3D OCT guidance**			**Metal overlapping**	**Metallic carina**	**Stent gap** **in SB** **ostium**
		**Recommendation**	**Optimal wiring**	**Final success**	**Recommendation**	**Optimal wiring**	**Final success**			
**One-stent**										
Crossover stenting	SB	Distal cell	50–66%	50–66%	Distal cell	87–100%	87–100%	no	In case of suboptimal wiring	None
**Two-stent**										
Culotte	MV	Distal cell	50–66%	<50%	Distal cell	87–100%	>80%	2-layer	In case of suboptimal wiring	Rare
	SB	Distal cell	50–66%		Distal cell	87–100%				
Crush	SB	Non-distal cell	>90%	>90%	Non-distal cell	>90%	>90%	2–3-layer	Inevitable	Rare
DK-Crush	SB	Non-distal cell	>90%	>80%	Non-distal cell	>90%	>80%	2–3-layer	Inevitable	Rare
	SB	Non-distal cell	>90%		Cell inside the hole made by first KBI	87–100%				
T-stenting	SB	Distal cell	50–66%	50–66%	Distal cell	87–100%	87–100%	no	Less or minimal	Sometimes
TAP	SB	Distal cell	50–66%	50–66%	Distal cell	87–100%	87–100%	2-layer	Inevitable	Rare

SB, side branch; MV, main vessel; DK-Crush, double kissing-Crush; KBI, kissing balloon inflation; TAP, T-stenting and minimal protrusion.

## Actionable recommendations

**Figure 9:**
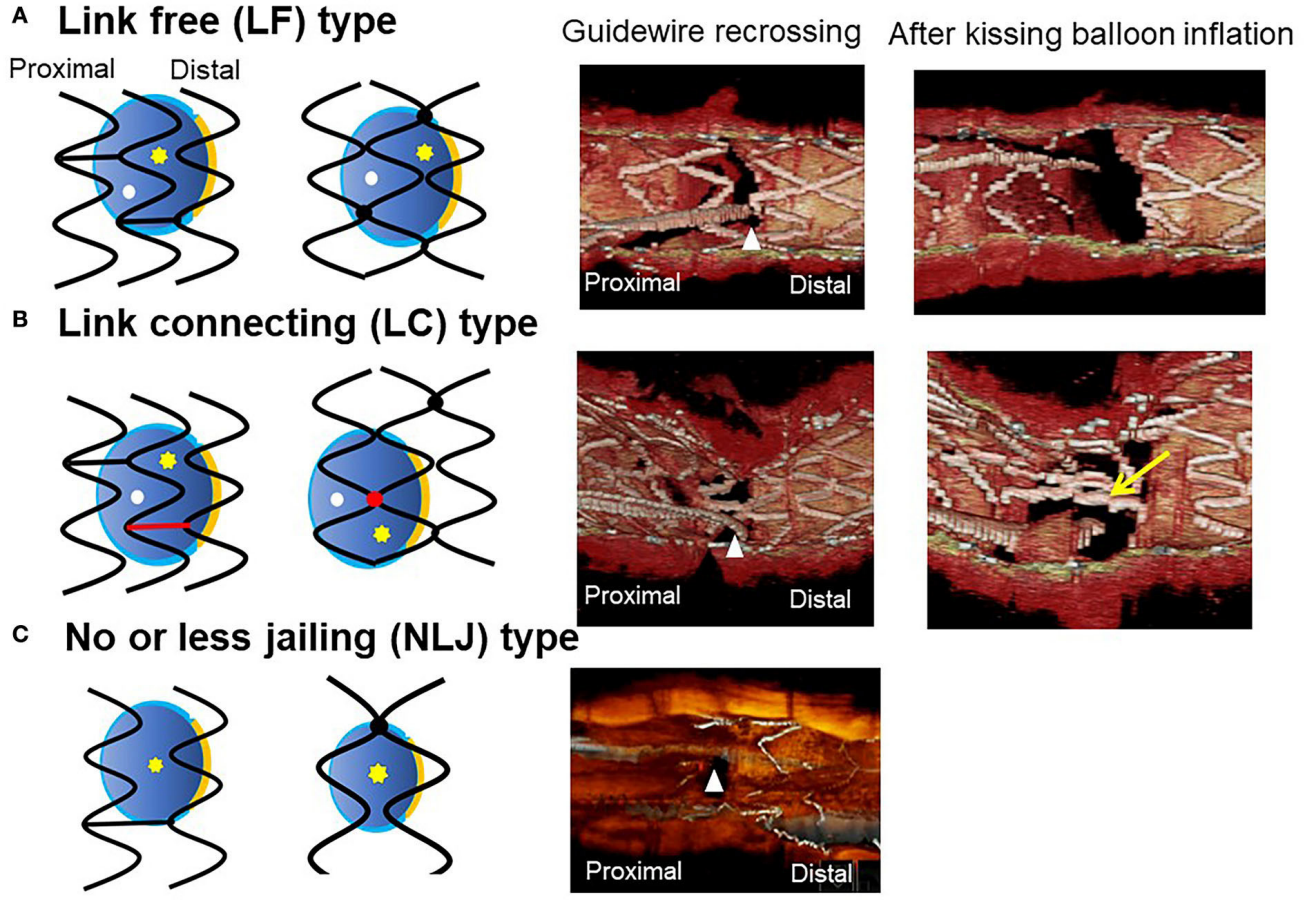
Classification of jailing strut configuration on the side branch ostium. In the scheme (left: in-phase type stent, right: out-of-phase type stent), orange line indicates the carina, red color indicates the link between hoops connecting to the carina, yellow asterisks, and white dots indicate the distal and proximal recrossing position. Middle column: 3D optical coherence tomography (OCT) image of guidewire recrossing point. Right column: 3D OCT image after final kissing balloon inflation (KBI). **(A)** Link-free (LF) type, **(B)** Link-connecting (LC) type, **(C)** No or less jailing (NLJ) type.

**Figure 10:**
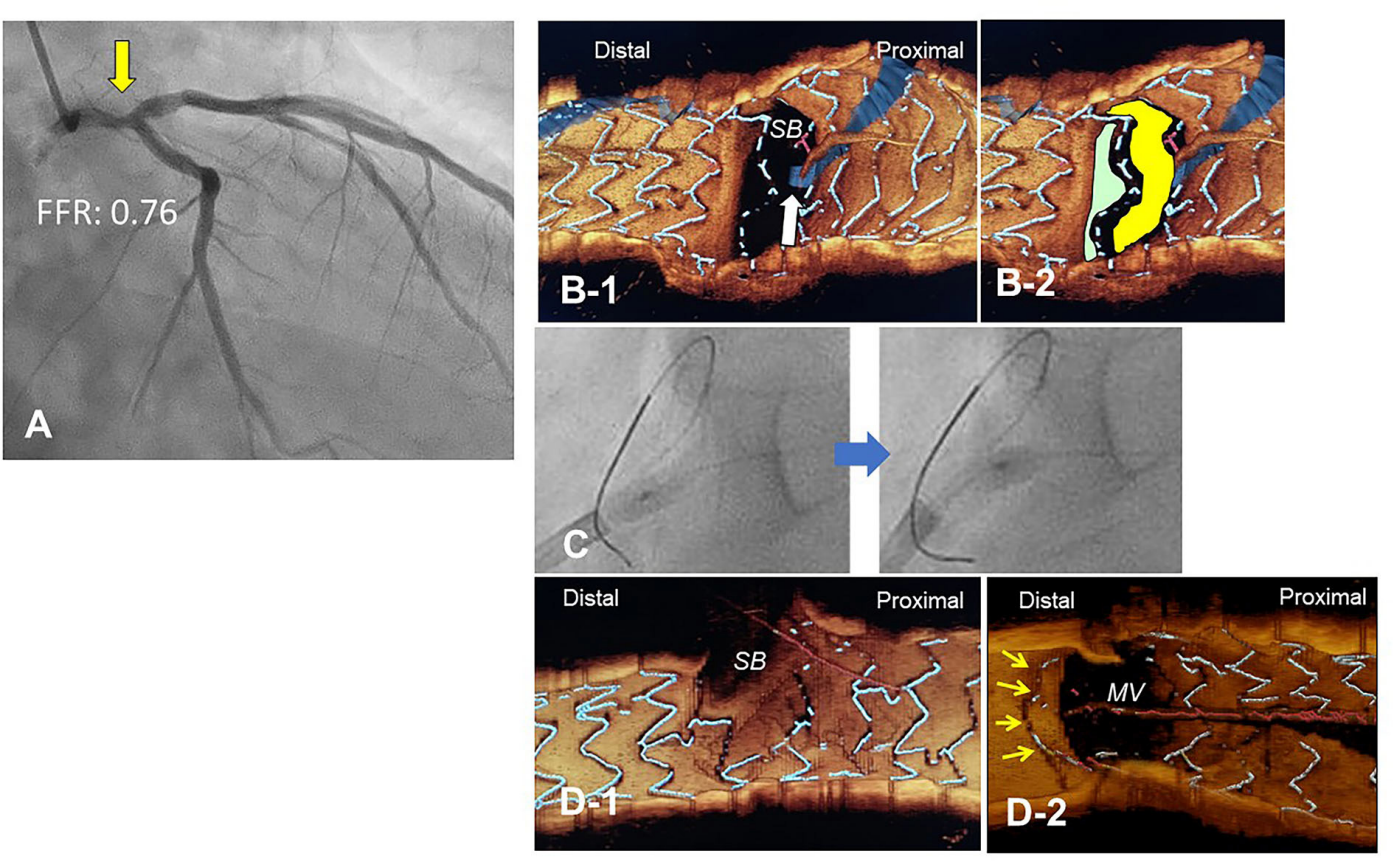
Push-fold method for complete removal of jailing struts in the side branch (SB) ostium. A 78-year-old man with Medina 1-1-0 lesion in LM bifurcation with a drop of fractional flow reserve (FFR) of 0.76 **(A)**. After cross-over stenting from LM to LAD with a Xience Alpine 3.5/28 mm stent and subsequent proximal optimization in LM with a 4.5 mm balloon, guidewire recrossing to the distal cell (green cell) was difficult even after several attempts and finally crossed to the proximal cell (yellow cell, white arrow) **(B)**. For the removal of the jailing struts in the SB ostium, an inflated 2.5/4 mm balloon was pushed from LM to LCX **(C)**. Final 3-D OCT demonstrated complete removal of the jailing struts in the SB ostium (**D-1**, pull back from main vessel [MV]), and the jailing strut folded toward the carina (**D-2**, pull back from SB).

The most contemporary concept of 3D OCT-guided SB treatment is based on the model of jailing strut configuration and guidewire recrossing cells. In the LF type, a higher success rate of optimal distal wiring can be achieved under 3D OCT guidance, which leads to optimal SB dilation (Figure 9). In the LC type, distal-cell wiring does not always reduce ISA (Figure 9). First, a more aggressive POT is used, which provides greater expansion of the bifurcation core and decreases the number of jailing struts. Changing to the NLJ type without SB compromise allows the SB to remain undilated. More protrusion into the SB ostium of the far-distal cell, which is neighboring distally to the distal cell with a link connection, allows the guidewire to recross to the far-distal cell without a link connection, leading to more SB ostial expansion. When these results are not obtained, guidewire recrossing to the cell that occupies most of the SB ostium and subsequent SB dilation are also reasonable treatments. Another cutting-edge treatment is the push-fold method, in which the guidewire is intentionally crossed in the proximal cell, and an inflated balloon pushes the jailing struts away in the direction of the SB ostium (34, 35) (Figure 10). In the NLJ type, SB treatment can be deferred unless severe compromise occurs (Figure 9).

## Discussion

**Figure 11:**
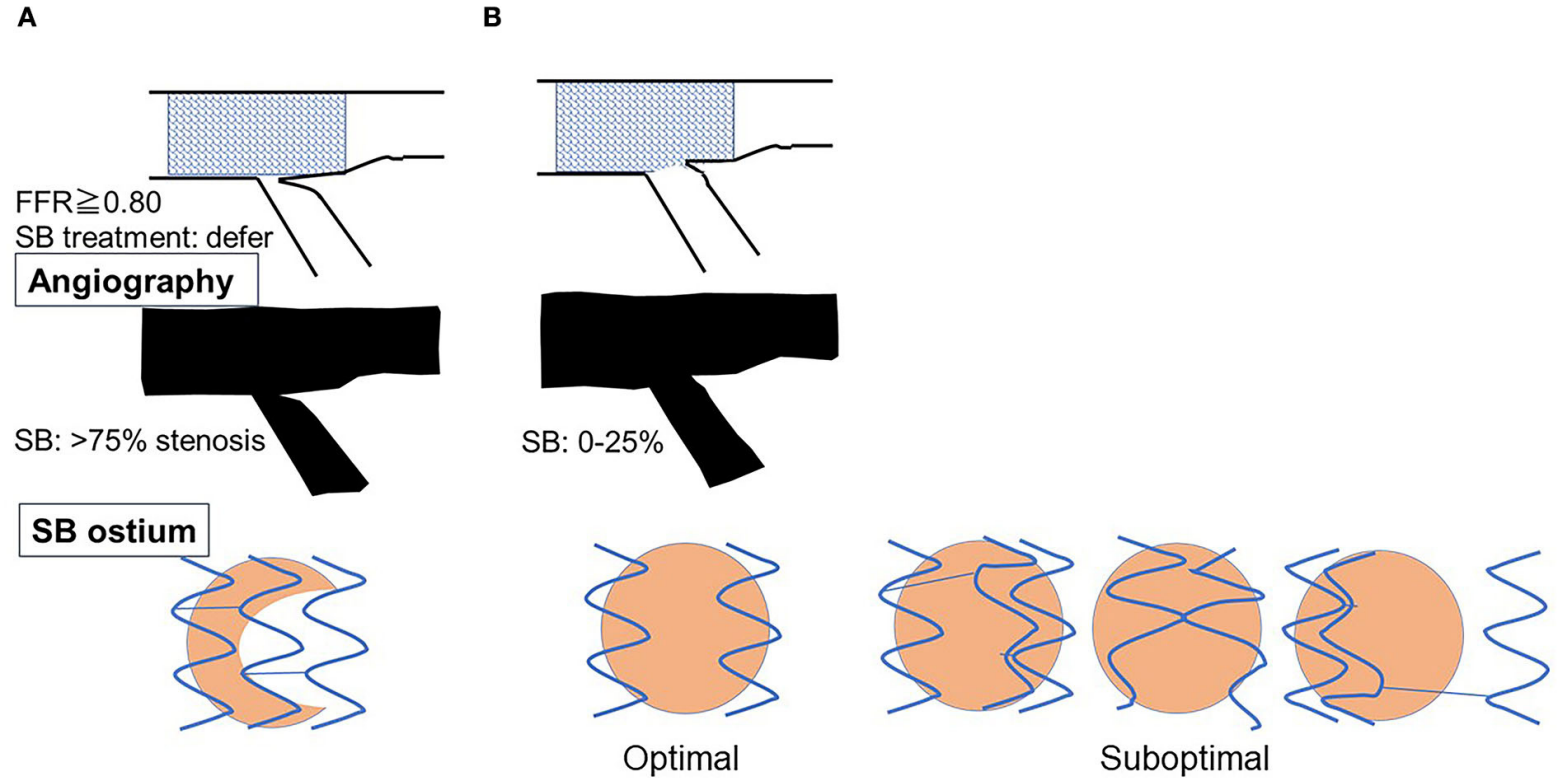
Scheme of side branch (SB) compromise after crossover stenting in the main vessel (MV) and subsequent SB dilation. **(A)** SB compromise after MV stenting with >75% stenosis on angiography and shifted carina (top and middle panels). When fractional flow reserve (FFR) is preserved at ≥0.80, it is recommended to defer SB treatment. The actual condition of the SB ostium is that the shifted carina narrows the SB ostium, which remains a sufficient area to not be physiologically significant stenosis, and the aligned struts jail the SB ostium (bottom panel). **(B)** Condition after SB dilation. In coronary angiography, SB stenosis is relieved with the correction of the shifted carina to the neutral position (top and middle panels). In the optimal treatment after optimal guidewire recrossing in link-free jailing strut configuration, ideal removal of jailing struts and adequate SB dilation are achieved (bottom left panel). However, suboptimal guidewire recrossing to the proximal cell or in the link-connecting type results in inadequate SB ostial dilation with clustering of jailing struts (bottom left panels). The success rate of optimal SB treatment is low (<50%) under angio-guidance, so dramatic improvement is feasible under 3D optical coherence tomography guidance.

**Figure 12:**
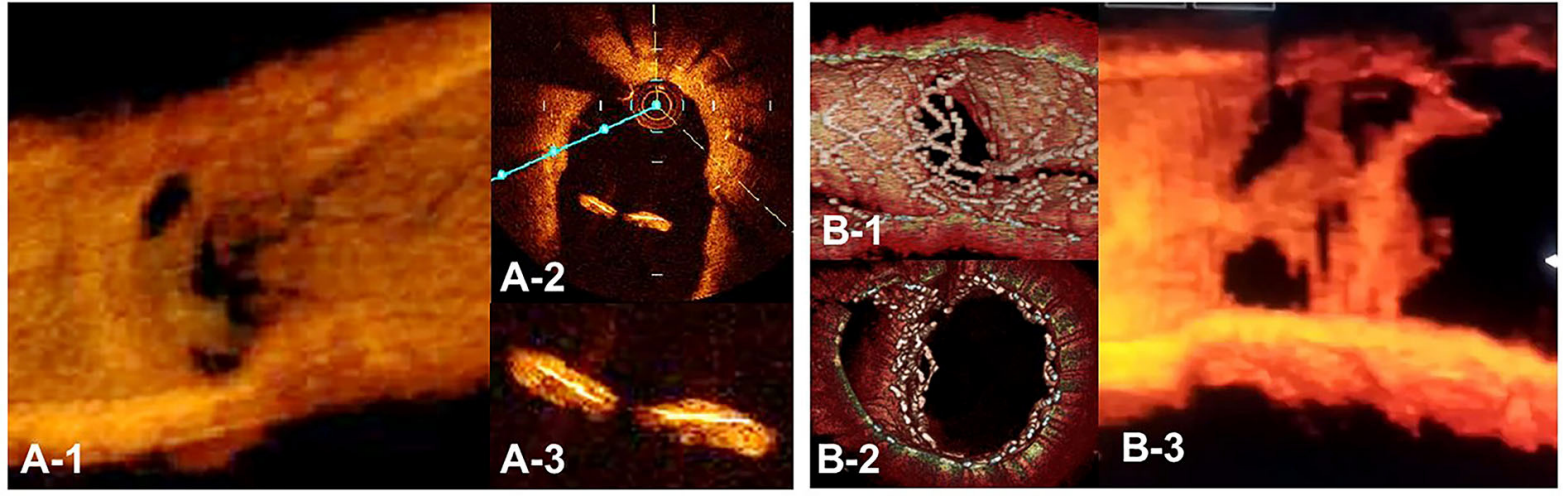
Optical coherence tomography **(**OCT) image of intimal proliferation on jailing struts at long-term follow-up period. A. One-year follow-up after crossover stenting with a synergy stent in the left anterior descending artery (LAD) without the fenestration of the diagonal branch. (**A-1)** 3D vessel view shows complete intimal coverage on the stent struts even in the jailing struts on the diagonal branch ostium with some luminal narrowing. **(A-2)** 2D imaging on the bifurcated site. **(A-3)** magnified view of the jailing struts. Complete intimal coverage of all surfaces of the struts was completed. **(B)** 3D OCT image after culotte stenting in LAD and diagonal branch using resolute integrity stents with significant metallic carina formation remaining (**B-1**: immediately after stenting, perpendicular view, **(B-2)** view from proximal to the bifurcation). **(B-3)** Two-year follow-up. 3D OCT image shows intimal coverage along the struts of the metallic carina, with several holes remaining (Supplementary Movie 13).

3D OCT imaging demonstrated that angio-guidance results in a lower success rate of optimal guidewire recrossing and a greater impact of link connection on the removal of jailing struts from the SB ostium than expected (4, 6). Ideal distal cell wiring in the LF type, which leads to optimal SB dilation with less ISA, was achieved in only 51% (54/105) of cases, even with 2D or 3D OCT guidance (4). In OPTIMUM, a randomized trial comparing 3D OFDI guidance and angio-guidance in coronary bifurcation stenting, angio-guidance produced more ISA than did 3D OFDI guidance (27.5 ± 14.2% vs. 19.5 ± 15.8%, *P* = 0.008) (6). Less optimal results in angio-guidance than expected may be one of the mechanisms for the failure of a conceptually perfect procedure. Discordance in the severity of SB stenosis after MV stenting between quantitative coronary angiography and physiological assessment of fractional flow reserve (FFR) is well-known, and FFR-guided SB treatment has been proposed to avoid unnecessary treatment in the SB with a high FFR value (36–38) (Figure 11A). However, computer simulation or bench testing of coronary flow in the strut-jailing or narrowed SB indicates turbulent flow behind the strut with low shear stress at the SB ostium (26, 39, 40), which might lead to stent thrombosis, restenosis, or fatal events (39, 41, 42). An ideal SB opening without SB stenosis or shifted carina indicates the restoration of optimal coronary flow circumstances in the bifurcation (Figure 11B, optimal treatment). Although the final KBI is expected to solve this issue, acceptable efficacy has not been found in several randomized trials and meta-analyses (19, 20). Angio-guided guidewire recrossing only was available in these clinical studies, in which more than half of the cases might be of suboptimal wiring or undesirable jailing strut configurations (LC type) for SB dilation (Figure 11B, suboptimal treatment). A long-term follow-up OCT study demonstrated more thrombus attachment and intimal proliferation on the jailing struts and narrowing of the SB ostium (40, 43–45) (Figure 12, Supplementary Movie 13). Elevation of the accuracy of guidewire recrossing to >90% under 3D OCT guidance and subsequent optimal SB dilation have great potential to improve the clinical outcome of provisional stenting. In 2-stenting, accurate guidewire recrossing to the optimal distal cell requires one time for the SB in systematic T-stenting and TAP, and two times for both MV and SB in culotte stenting. Therefore, the low performance of angiography-guided guidewire recrossing might weaken the clinical outcome of these stentings; in particular, the success rate of ideal optimal guidewire recrossing in both MV and SB (50–66% in each branch) is estimated to be <50% in culotte stenting, which might be one reason that its significant advantage over other 2-stentings has not been reported (46–48) except for TAP stenting (49) regardless of excellent performance in bench testing (27). The risk of restriction of MV stent expansion (Figure 5) is listed as a significant disadvantage compared to DK-crush stenting (29, 46, 50) with a higher probability of suboptimal guidewire recrossing or undesirable jailing strut pattern (LC type). Since some metallic carina formation is allowed in crush stenting, strict guidewire recrossing to the SB is not required except for abluminal wiring outside of the SB stent. Although guidewire recrossing before final KBI is easier than crush stenting, wiring to outside of the hole created by first KBI should not be done to avoid further deformation of the jailing struts in DK-crush stent. Therefore, accurate assessment of guidewire recrossing in the optimal cell and monitoring of the stent configuration during the procedure under 3D OCT imaging guidance is more essential in complex 2-stenting.

As for the limitation of using 3D OCT imaging guidance in the bifurcation intervention, it requires more OCT observations due to the strict detection of the procedural failure, which is likely to result in more consumption of contrast medium, procedural time, and radiation dose. Although excellent detection of critical stent failure leading to adverse cardiac events has been reported in clinical studies, its efficacy on long-term clinical outcomes over angio-guidance has not been clearly demonstrated.

In conclusion, 3D OCT imaging guidance in coronary bifurcation intervention is feasible with LMWD substituted for contrast medium. Its clear visualization facilitates monitoring of guidewire recrossing points and stent configuration during and after complex procedures, which promotes optimal treatment more accurately than angio-guidance.

## Author contributions

YM conceived the study and drafted and reviewed the manuscript.

## Conflict of interest

The author declares that the research was conducted in the absence of any commercial or financial relationships that could be construed as a potential conflict of interest.

## Publisher's note

All claims expressed in this article are solely those of the authors and do not necessarily represent those of their affiliated organizations, or those of the publisher, the editors and the reviewers. Any product that may be evaluated in this article, or claim that may be made by its manufacturer, is not guaranteed or endorsed by the publisher.

## Supplementary material

Supplementary Movie 1 Comparison of image quality in frequency-domain optical coherence tomography.

Supplementary Movie 2 Flythrough view of three-dimensional optical coherence tomography indicating guidewire recrossing to the side branch.

Supplementary Movie 3 **(A)** Baseline coronary angiography. **(B)** Final coronary angiography.

Supplementary Movie 4 **(A)** Baseline coronary angiography. **(B)** Final coronary angiography.

Supplementary Movie 5 Three-dimensional optical coherence tomography images indicating guide wire recrossing points in first, second, and third attempts.

Supplementary Movie 6 **(A)** Baseline coronary angiography. **(B)** Final coronary angiography.

Supplementary Movie 7 Confirmation of guidewire recrossing point by three-dimensional optical coherence tomography imaging.

Supplementary Movie 8 Two- and three-dimensional optical coherence tomography images after final procedure.

Supplementary Movie 9 **(A)** Baseline coronary angiography. **(B)** Final coronary angiography.

Supplementary Movie 10 Three-dimensional optical coherence tomography images during each step in double kissing crush stenting.

Supplementary Movie 11 **(A)** Baseline coronary angiography. **(B)** Final coronary angiography.

Supplementary Movie 12 Three-dimensional optical coherence tomography images indicating guidewire recrossing points.

Supplementary Movie 13 Three-dimensional optical coherence tomography image of metallic carina with luminal narrowing 2-year after culotte stenting.
